# ARDS and corticosteroids: beyond COVID-19

**DOI:** 10.1186/s41479-026-00200-7

**Published:** 2026-05-25

**Authors:** Mariano Esperatti, Matías Olmos, Nora Fuentes

**Affiliations:** 1 Intensive Care Department, Hospital Privado de Comunidad, Cordoba 4545, Mar del Plata, 7600 Argentina; 2 Facultad de Medicina, Universidad Nacional de Mar del Plata, Mar del Plata, Argentina; 3 Consejo Nacional de Investigaciones Científicas y Técnicas (CONICET), Buenos Aires, Argentina

**Keywords:** Acute respiratory distress syndrome, Corticosteroids, Inflammation, Sepsis, Pneumonia, COVID‑19, Precision medicine

## Abstract

Acute respiratory distress syndrome (ARDS) remains a high‑mortality condition despite major advances in ventilatory and supportive care. Because lung injury is driven by uncontrolled inflammation and disruption of the alveolar–capillary barrier, corticosteroids have long been considered a biologically plausible therapy. Over several decades, randomized trials have evaluated different corticosteroid types, doses, durations, and initiation timings in heterogeneous populations, including patients with primary ARDS and those with sepsis or pneumonia complicated by ARDS. The COVID‑19 pandemic provided a unique opportunity to study a more homogeneous cause of ARDS, generating additional evidence supporting corticosteroid efficacy in virus‑related respiratory failure. Despite these advances, clinical practice remains variable worldwide. Evolving definitions, diverse trial designs, and etiologic variability have led to uncertainty regarding indication, optimal dosing, drug selection, and treatment duration. The new global definition of ARDS now includes patients supported with noninvasive ventilation or high‑flow nasal oxygen, raising questions about how evidence derived from invasively ventilated populations applies to these groups. Parallel research identifying inflammatory subphenotypes with potentially divergent treatment responses further underscores the need for individualized, evidence‑based strategies. In this review, we examine conceptual, biological, and clinical considerations and synthesize the available evidence to inform decision‑making. We propose a pragmatic, context‑based framework to guide corticosteroid use in ARDS according to etiology and patient characteristics, emphasizing early initiation—ideally within 24–48 hours—and regimens equivalent to ≥80 mg/day of methylprednisolone or ≥400 mg/day of hydrocortisone for at least seven days, which have demonstrated efficacy with an acceptable safety profile. Research priorities include optimizing dose and duration, evaluating non‑ventilated and underrepresented subgroups, and clarifying phenotype‑specific effects and long‑term safety.

## Background

The human lung has a limited capacity to respond to acute injury. Pulmonary infections, trauma, and extrapulmonary insults associated with systemic inflammation may converge into a final common pathway—acute inflammation of the lung parenchyma [1]. Acute respiratory distress syndrome (ARDS) is a conceptual construct that integrates the diverse clinical and pathophysiological manifestations of this process [2]. It is characterized by inflammatory alveolar edema due to increased epithelial and endothelial permeability, loss of aerated tissue, elevated lung elastance, and impaired gas exchange [3].

Although its definition has evolved, three core features remain consistent: acute onset, characteristic radiographic findings, and varying degrees of hypoxemia [3]. The most recent global definition, derived from the Berlin criteria, preserves these key components—acute respiratory failure within seven days not fully explained by cardiac dysfunction, oxygenation impairment (PaO₂/FiO₂ <300), and bilateral pulmonary infiltrates—while broadening eligibility to all modes of ventilatory support (invasive or noninvasive positive‑pressure ventilation and high‑flow nasal oxygen), allowing oxygenation assessment via SpO₂/FiO₂, and recognizing infiltrates identified by chest radiography, computed tomography, or lung ultrasound [4].

Historically, ARDS has carried a high mortality burden. From its description in the late 1960s through the mid‑1990s, mortality remained approximately 50–60%, unchanged despite evolving diagnostic criteria [5, 6]. Since the late 1990s, and especially after 2000, widespread adoption of lung‑protective ventilation and conservative fluid strategies has progressively reduced mortality [7, 8]. The introduction of prone positioning for moderate‑to‑severe ARDS further improved survival, although these interventions remain inconsistently implemented in daily practice. Current mortality still ranges from 30% to 40%, depending on disease severity [9, 10].

Regardless of its precipitating cause or etiologic heterogeneity, ARDS exemplifies acute alveolar–capillary injury in which inflammation is the central pathogenic mechanism. This has long spurred interest in immunomodulatory therapies—particularly corticosteroids—whose pleiotropic effects on immune regulation, vascular permeability, and tissue repair provide a strong biological rationale for potential benefit. Yet, heterogeneity of the syndrome and methodological variability among trials have yielded inconsistent results, sustaining ongoing debate regarding their therapeutic role [11, 12]. Real-world data likewise show heterogeneous practice, with corticosteroid use across ARDS severity categories ranging from 18% to 40% globally [9, 10].

The objective of this review is to critically appraise the rationale for corticosteroid therapy in ARDS and to synthesize the available clinical evidence to guide individualized treatment decisions according to disease etiology, biological context, and patient characteristics, while highlighting current knowledge gaps and priorities for future research.

## Biological rationale for corticosteroid therapy in ARDS

ARDS can be understood as an exaggerated pulmonary inflammatory response to an initial insult. Dysregulated activation of transcription factors such as nuclear factor kappa B (NF‑κB) and pro‑inflammatory cytokines—including tumor necrosis factor‑alpha (TNF‑α), interleukin‑1 beta (IL‑1β), and interleukin‑6 (IL‑6)—initiates and perpetuates lung injury, ultimately contributing to multiorgan failure [13, 14]. Within the lung, this process results in diffuse alveolar damage, protein‑rich interstitial and alveolar edema, loss of alveolar–capillary barrier integrity, and migration of inflammatory cells into the airspaces [15].

This sustained dysregulated response also contributes to critical illness–related corticosteroid insufficiency (CIRCI), encompassing hypothalamic–pituitary–adrenal axis dysfunction, inadequate adrenal cortisol production relative to demand, and tissue resistance to glucocorticoids [16]. Importantly, these alterations extend beyond hormonal imbalance to include reduced activity of the glucocorticoid receptor‑α (GRα) and mitochondrial dysfunction, both of which perpetuate inflammation and impair restoration of homeostasis [16–18]. If the acute phase is survived, tissue regeneration may ensue; however, persistent low‑grade inflammation can contribute to post‑ICU syndrome and increased long‑term morbidity and mortality [19].

Within this pathophysiologic framework, endogenous (cortisol) and exogenous corticosteroids (methylprednisolone, hydrocortisone, dexamethasone) exert pleiotropic effects across interconnected mechanisms. At the genomic level, they modulate transcription of pro‑ and anti‑inflammatory genes; at the non‑genomic level, they trigger rapid responses via membrane‑associated receptors; and at the mitochondrial level, they enhance biogenesis, optimize energy metabolism, and regulate adaptive signaling pathways [20, 21]. Far from being independent phenomena, these actions converge through the GRα, whose proper function orchestrates adaptive programs that allow the transition from uncontrolled inflammation to resolution, tissue repair, and restoration of homeostasis [16–18].

When administered over a prolonged period and at physiological doses, corticosteroids act beyond correcting relative adrenal insufficiency in critical illness. Under GRα regulation, they sequentially modulate genomic and non‑genomic programs governing the host response: attenuating early pro‑inflammatory activation, enhancing anti‑inflammatory mediators, promoting mitochondrial‑driven tissue regeneration and differentiation, and preventing persistence of a pathological inflammatory state during the resolution and recovery phases. In doing so, corticosteroids not only compensate for relative insufficiency but also restore the immune and tissue capacity to progress in an orderly fashion through inflammation, resolution, and repair—yielding potential benefits in the acute course and in long‑term recovery [16–18, 22].

This biological rationale underpins therapeutic use of corticosteroids in ARDS, aiming not only to suppress inflammation but also to re‑establish physiological resilience and promote recovery.

## Definitional and etiological intersections in ARDS: relevance to corticosteroid therapy

When considering corticosteroids in ARDS, several general and specific issues related to each clinical scenario should be taken into account. The structural substrate that defines ARDS is diffuse alveolar damage (DAD), which progresses through an exudative phase—dominated by edema and alveolar collapse—followed by proliferative and, eventually, fibrotic stages [22, 23].

Although DAD represents the histopathologic hallmark of ARDS, autopsy studies demonstrate highly variable findings among patients meeting clinical ARDS definitions. Depending on disease stage, only 12–58% of these patients present DAD, while others display alternative diagnoses such as pneumonia, atelectasis, or even an absence of specific lesions [24]. This variability reflects that ARDS is a clinical and conceptual construct—defined by clinical criteria, encompassing multiple etiologies, and affecting heterogeneous patients. Consequently, a salient feature of the syndrome is its marked clinical and biological heterogeneity, which may account for the diverse responses observed with different therapeutic strategies [25, 26].

Although ARDS is often categorized as resulting from infectious or non-infectious insults, the lung is not sterile. Alterations in pulmonary microbiome composition have been described in ARDS and have been associated with clinical outcomes [27]. However, whether these changes represent causal mechanisms of lung injury or secondary phenomena of critical illness remains uncertain, and their therapeutic implications are not yet established.

ARDS is dynamic. Within the first hours of exposure to positive‑pressure ventilation, a proportion of patients may no longer meet ARDS criteria or may shift to a different severity category [28]. Conversely, up to 20% of patients with acute respiratory failure requiring positive‑pressure ventilation and unilateral infiltrates may later develop bilateral opacities, thus fulfilling ARDS criteria at a later stage [29, 30].

Worldwide surveys show that the leading cause of ARDS is severe pneumonia (≈60%), followed by extrapulmonary sepsis (≈16%) and aspiration (≈15%). Trauma, pulmonary contusion, transfusion, inhalation injury, and pancreatitis are far less common [5, 6]. Notably, severe pneumonia and sepsis/septic shock are conditions for which robust evidence supports systemic corticosteroids [31], and severe pneumonia alone accounts for approximately half of all sepsis and septic shock cases [32]. Thus, substantial etiological overlap among ARDS, severe pneumonia, and sepsis underscores the complexity of interpreting trial evidence, as most studies focus on specific diagnostic categories without fully accounting for the continuum that often exists among these syndromes.

## Corticosteroids in Non‑COVID‑19 ARDS

The clinical evidence regarding the efficacy and safety of corticosteroids in ARDS can be divided into two broad phases: the pre‑pandemic era and the COVID‑19 period. During the four decades preceding the pandemic, evidence accumulated through several randomized controlled trials conducted at various historical periods [32–41]. When translating this evidence into practice, it is crucial to acknowledge that each trial was performed under distinct ARDS definitions, standards of care, and co‑interventions reflecting the critical‑care practices of its time.

With the emergence of COVID‑19, an overwhelming body of evidence was generated in a short timeframe. In two to three years, randomized trials of systemic corticosteroids enrolled more patients than all prior ARDS trials conducted over the preceding decades [42–47]. Following the pandemic, however, no additional large‑scale ARDS corticosteroid trials have been reported.

Table 1 summarizes the principal RCTs evaluating corticosteroids in non‑COVID‑19 ARDS [32–41]. These studies were heterogeneous in design, patient populations, dosing regimens, treatment durations, and outcomes. Importantly, all enrolled patients were receiving invasive mechanical ventilation at baseline. Two trials specifically examined “persistent ARDS” (after day seven of onset) using methylprednisolone for 21–28 days [40, 41]. In the larger of these, although overall mortality did not differ, several secondary outcomes favored corticosteroids—more ventilator‑free and organ‑failure‑free days. However, patients who initiated treatment after day 14 had higher 60‑day mortality [41], aligning with the biological rationale that therapy should be administered early rather than late.

**Table 1:** Randomized controlled trials evaluating the efficacy and safety of corticosteroids versus placebo in patients with non-COVID-19 ARDS

Study	Total Poulation (n) [I:Intervention (n), C:Control (n)]	Main inclusion criteria	PaO2/FiO2 (mmHg.) at inclusion, I:intervention(n) C: Control (n)	Pneumonia as main cause of ARDS, n (%)	Steroid Type/dosage schedule	Duration of treatment (d)	Eficacy, Mortality Intervention (I), and Control (C)	Eficacy, Other outcomes, (Intervention vs control group)	Safety, Main adverse outcomes (Intervention vs control group)
Heming et al. (post-hoc-analysis). 2024	648 (I = 320 C = 328)	Septic Shock + ARDS (Berlin criteria)	I = 176 C = 179	337 (52)	Hydrocortisone 50 mg every 6 h, plus fludrocortisone 50 mcg/d	7	I = 48% C = 57% (OR = 0.85) (day 90)	*VFDs: not reported *IMV duration: not reported *ICU LOS: not reported *Hosp LOS: not reported	Hyperglycemia: No difference Infections: no difference Weakness: no differences
Villar et al. 2020	277 (I = 139 C = 138)	Berlin moderate-to-severe ARDS und,er IMV ≥ 24 h (PEEP ≥ 10 cmH₂O, FiO₂ ≥0.5)	I = 142 C = 143	177 (53)	Dexamethasone IV, 20 mg once daily from day 1 to day 5, 10 mg once daily from day 6 to day 10 (or until intubation)	10	I = 21% C = 36% (*p* = 0.004)	↑ VFDs ↓IMV (D) ↓ICU and Hospital LOS	Hyperglycemia: No difference Infections: no differences Weakness: Not reported
Tongyoo, et al. 2016	197 (I = 98 C = 99)	Sepsis/septic shock + ARDS (Berlin definition) under IMV	I = 175 C = 172	100 (51)	Hydrocortisone 50 mg q 6 h,	7	I = 22% C = 27% p = 0.043 (day 28)	VFDs: no difference IMV duration: no difference ICU/Hosp LOS: not reported Faster improvement in LIS and PaO₂/FiO₂	Hyperglycemia: Higher (intervention group) Infections: no differences Weakness: not reported
Rezk, et al. 2013	27 (I = 18 C = 9)	PaO2/FiO2 < 200, PAWP < 18 mmHg., IMV, bilateral pulmonary infiltrataes (first 48 h)	I = 77 C = 113	19 (70%)	Methylprednisolone loading dose 1 mg/kg, then an infusion of 1 mg/kg/d from d 1 ↓↓to 14; 0.5 mg/kg/d days 15–21; 0.25 mg/kg/d days 22–25; and 0.125 mg/kg/d days 26–28	28	I = 0% C = 33% (*p* = 0.009) (day 14)	VFDs: not reportted ↓IMV (d) ICU/Hosp LOS: not reported	Hyperglycemia: Not reprted Infections: lower in intervention group Weakness: Not reported
Sabry et al., 2011	80 (I = 40 C = 40)	Severe community-acquired pneumonia (ATS criteria, PaO₂/FiO₂ <250 + bilateral infiltrates)	I = 178 C = 182	80 (100)	Hydrocortisone loading dose 200 mg, then an infusion of 180 mg/d	7	I = 5% C = 15% p = 0.66 (at ICU discharge)	VFDs: not reported IMV duration: not reported ICU/Hosp LOS: not reported	Hyperglycemia: Not reprted Infections: no difference Weakness: not reported
Meduri et al. (2007)	91 (I = 63 C = 28)	American European Consensus definition criteria under IMV, dentro de las 72 h de diagnostico	I = 118 C = 125	38 (42%)	Methylprednisolone loading dose 1 mg/kg, then an infusion of 1 mg/kg/d from d 1 to 14; 0.5 mg/kg/d days 15–21; 0.25 mg/kg/d days 22–25; and 0.125 mg/kg/d days 26–28	28	I = 24% C = 43% (*p* = 0.07)	↑VFDs ↓IMV ↓ICU LOS: ↓Hosp LOS	Hyperglycemia: Higher Infections: no differences Weakness: higher
Annane, et al. (post-hocanalysis). (2006)	177 (I = 85 C = 92)	Septic shock-associated early ARDS: bilateral, PaO2/FIO2 < 200 mm Hg, no evidence of left atrial hypertension.	I = 104 C = 108	106 (60)	Hydrocortisone 50 mg every 6 h, plus fludrocortisone 50 mcg/d	7	I = 58% C = 67% p = 0.043 (day 28)	VFDs: no difference IMV duration: not reported ICU/Hosp LOS: not reported	Hyperglycemia: Not reported Infections: no differences Weakness: not reported
ARDS Clinical Trials Network. (2006)	180 (I = 89 C = 91)	ARDS (7–28 days after onset) PaO2/FIO2 < 200	I = 126 C = 126	76 (42%)	Methylprednisolone loading dose 2 mg/kg, then 2 mg/kg/d days 1–14; 1 mg/kg/d days 15–21;	21	Overall I = 29% C = 29% (*p* = 1.00) ARDS onset > 14d I = 35% C = 8% (*p* = 0.02)	↑VFDs ↓IMV (d) ↓ICU LOS Hosp LOS: no differences	Hyperglycemia: Not reported Infections: no differences Weakness: Not reported
Confaloniri, et al. 2005	46 (I = 23 C = 23)	Severe Community acquired pneumonia. ATS criteria of severe pneumonia, including Pao2/Fi02 < 250 and bilateral infiltrates	I = 141 C = 178	46 (100%)	Hydrocortisone loading dose 200 mg, then an infusion of 240 mg/d	7	I = 0% C = 38% *p* = 0.001 (day 60)	↑VFDs:higher ↓IMV (d) ↓ICU/Hosp LOS	Hyperglycemia: Not reported Infections: no differences Weakness: no differences
Meduri et al. (1998)	24 (I = 16 C = 8)	AECC ARDS within 72 h of diagnosis under IMV	I = 161 C = 141	15 (62%)	Methylprednisolone loading dose 2 mg/kg, then 2 mg/kg/d days 1–14; 1 mg/kg/d days 15–21; 0.5 mg/kg/d days 22–28; 0.25 mg/k/d days 29–30	25	I = 13% C = 63% (*p* = 0.03)	VFDs: not reported ↓IMV (d) ICU LOS: not reported Hosp LOS: not reported	Hyperglycemia: No difference Infections: no differences Weakness: Not reported

*All outcomes were reported for patients with ARDS and community-acquired pneumonia (*n* = 337). Subjects receiving corticosteroids showed increased ventilator-free days and longer ICU/hospital LOS. Results were not reported for ARDS without pneumonia (*n* = 298)

Abbreviations. ARDS = acute respiratory distress syndrome; AECC = American–European Consensus Conference; ATS = American Thoracic Society; CAP = community-acquired pneumonia; FiO₂ = fraction of inspired oxygen; IMV = invasive mechanical ventilation; IV = intravenous; LD = loading dose; LIS = lung injury score; LOS = length of stay; NR = not reported; PaO₂ = arterial oxygen pressure; PAWP = pulmonary artery wedge pressure; PEEP = positive end-expiratory pressure; q6h = every 6 hours; VFDs = ventilator-free days

The remaining trials shared common features: corticosteroids were started early (within 24–48 hours of ARDS onset), doses were low to intermediate [21], and pneumonia was the most frequent precipitating cause—reflecting real‑world epidemiology [9]. Overall, these studies demonstrated either a consistent trend toward, or a statistically significant improvement in, mortality and other clinically relevant outcomes, while adverse events were infrequent or within acceptable thresholds.

Meta‑analyses of randomized trials in non‑COVID-19 ARDS confirmed a reduction in mortality, with pooled risk ratios ranging from 0.71 to 0.84 across analyses [26, 48]. This beneficial effect persisted when only studies meeting the Berlin definition were included [48]. Corticosteroids were also associated with shorter durations of mechanical ventilation (median reduction ~4 days) and reduced hospital length of stay (median reduction ~8 days) [48]. In terms of safety, corticosteroids did not significantly influence gastrointestinal bleeding (RR 1.08; 95% CI 0.87–1.34) or secondary infections (RR 0.97; 95% CI 0.89–1.05). Neuromuscular weakness (RR 1.22; 95% CI 1.03–1.45; absolute risk increase 1.4%; low certainty) and neuropsychiatric complications (RR 1.19; 95% CI 0.82–1.74) did not appear substantially increased. Hyperglycemia (RR 1.21; 95% CI 1.11–1.31) and a probable increase in hypernatremia were consistently observed [49].

### Corticosteroids in ARDS associated with sepsis

Among patients with sepsis and vasopressor requirement, the efficacy and safety of corticosteroids have been extensively investigated. In this group, corticosteroids lower both short‑ and long‑term mortality (RR 0.94; 95% CI 0.89–1.00; and RR 0.93; 95% CI 0.88–0.98, respectively). Accordingly, international guidelines—including those from the Society of Critical Care Medicine—recommend systemic corticosteroids for patients with septic shock requiring vasopressors [31].

Four major RCTs enrolling > 5,800 patients evaluated corticosteroids in septic shock [50–53]. Two used hydrocortisone 50 mg every six hours plus fludrocortisone 50 μg daily for seven days and demonstrated significant mortality benefit [50–53]. The other two—using identical hydrocortisone regimens but without fludrocortisone—did not show mortality reduction, although they shortened shock duration [50–53]. Most participants (>95%) were mechanically ventilated, and the majority had PaO₂/FiO₂ <300, suggesting frequent coexistence of lung injury.

Two of these trials analyzed outcomes in patients with concomitant ARDS. In Annane et al., about 60% met ARDS criteria; among non‑responders to cosyntropin, 28‑day mortality was lower with corticosteroids (42% vs 67%; HR 0.57; 95% CI 0.36–0.89), whereas no difference appeared in patients without ARDS [38]. In a prespecified subgroup of APROCCHSS, hydrocortisone plus fludrocortisone reduced 90‑day mortality in ARDS (48% vs 57%; OR 0.72; 95% CI 0.53–0.98); among patients without ARDS, mortality was 36% vs 40% (OR 0.85; 95% CI 0.61–1.20) [32]. Together, these findings support a survival advantage with corticosteroids in ARDS secondary to sepsis requiring vasopressors, with the most reproducible benefit seen with hydrocortisone plus fludrocortisone for at least seven days.

### Corticosteroids in ARDS associated with pneumonia

In community‑acquired pneumonia (CAP), corticosteroids are recommended for severe disease, with reductions in hospital mortality (RR 0.62; 95% CI 0.45–0.85), need for mechanical ventilation, and length of stay [31]. This mortality effect appears larger than in sepsis and was not observed in less severe pneumonia. In most trials, severity was defined by the Pneumonia Severity Index or ATS criteria [54]. Many—but not all—participants met ARDS criteria.

Among patients with severe pneumonia requiring invasive mechanical ventilation, up to one‑third fulfill ARDS definitions, and their prognosis approximates that of non‑pneumonia ARDS [55]. Although dedicated trials in “pneumonia plus ARDS” are lacking, sensitivity analyses from pneumonia meta‑analyses have not shown effect modification by the underlying population (ARDS, pneumonia, or sepsis) [56].

Pneumonia, on the other size, accounts for roughly one‑half to two‑thirds of septic shock cases in which corticosteroids are effective. In APROCCHSS trial, among patients with pneumonia, mortality was 39% with corticosteroids vs 51% with placebo (OR 0.60; 95% CI 0.43–0.83), whereas in sepsis from other sources mortality was 46% vs 48% [32].

The CAPE COD trial evaluated hydrocortisone infusion (200 mg/day for up to seven days or until ICU discharge) in severe CAP without shock, reducing 28‑day mortality from 12% to 6% [57]. Most participants met severity based on respiratory failure, as vasopressors were excluded. Notably, 44% were on positive‑pressure ventilatory support (median PaO₂/FiO₂ 172), and 42% were on high‑flow nasal oxygen (median PaO₂/FiO₂ 130). All had pulmonary infiltrates. Thus, more than 40% likely met the Berlin definition of ARDS, and over 80% would fulfill the global ARDS definition. Taken together, these data suggest a mortality benefit of corticosteroids in pneumonia‑associated ARDS, possibly greater than in other etiologies. The most consistently supported regimens are hydrocortisone (with fludrocortisone when shock is present) for at least seven days.

Currently, no randomized trials have specifically evaluated the efficacy or safety of corticosteroids in hospital-acquired or ventilator-associated pneumonia, nor in ARDS explicitly secondary to these etiologies. Available evidence specific to ICU-acquired pneumonia derives from observational studies, which have suggested a potential association between corticosteroid exposure and worse outcomes in selected subgroups, particularly among patients with HAP, as opposed to VAP, and among those without concomitant ARDS; no such association was observed in patients with ARDS [58]. Accordingly, caution may be warranted when considering corticosteroids in these populations. In the absence of dedicated randomized evidence, corticosteroid use should otherwise follow general ARDS-based recommendations

With the evidence currently available, corticosteroids cannot be recommended for use with the primary intent of preventing ARDS. Earlier preventive trials, conducted predominantly in the 1980s and using high-dose bolus methylprednisolone regimens, did not demonstrate benefit and suggested possible harm [59]. More recent randomized trials in selected high-risk populations—such as severe trauma and traumatic brain injury—have evaluated low-dose hydrocortisone-based regimens to prevent hospital-acquired pneumonia, with evidence of reduced ventilator-associated pneumonia in some settings [60, 61]. However, these trials were neither designed to prevent pneumonia-induced ARDS nor did they demonstrate a reduction in ARDS incidence.

### Corticosteroids in ARDS from other causes

Fewer than 25% of ARDS cases arise from causes other than pneumonia or sepsis, including aspiration, trauma‑related lung injury, pancreatitis, or transfusion‑related reactions. These populations have been under‑represented in RCTs of corticosteroids in ARDS, and no trial has targeted these etiologies specifically. Recommendations therefore rely on indirect evidence from subgroup analyses of meta‑analyses, which have not demonstrated significant subgroup effects—suggesting that benefits observed in overall ARDS populations may apply regardless of etiology [26, 31, 48].

For transfusion‑related acute lung injury (TRALI), ARDS typically resolves rapidly—usually within one to three days, with a mean time to resolution of ~1.4 days in observational studies [62]. Given this favorable prognosis and lack of evidence for steroid benefit, when transfusion is the most likely cause it is prudent to observe for 24–48 hours before initiating corticosteroids or to discontinue therapy if rapid resolution occurs.

Severe pancreatitis is an important non‑infectious cause of ARDS [63]. No RCTs have specifically evaluated corticosteroids in pancreatitis‑associated ARDS. Small trials of corticosteroids in acute pancreatitis—not limited to ARDS—have suggested reduced organ failure, fewer surgical interventions, and possibly decreased mortality, but are limited by small samples and methodological weaknesses [64]. Until more robust evidence is available, applying general ARDS principles appears reasonable for these patients.

## Corticosteroids in COVID‑19 ARDS

SARS‑CoV‑2 is the etiologic agent of COVID‑19 [65]. Although most infections are asymptomatic or mild, a subset develops acute respiratory failure and ARDS [66, 67]. At the onset of the pandemic, debate surrounded whether COVID‑19 ARDS differed from traditional forms. Clinically, some patients showed profound hypoxemia with relatively preserved respiratory compliance early in illness, leading to proposed “L” and “H” phenotypes and concerns about delayed intubation in selected cases [68, 69].

Pathologically, vascular involvement appears more pronounced than in other ARDS etiologies. Autopsy and imaging studies described widespread microthrombosis, vasculopathy, and pulmonary vascular congestion, contributing to gas‑exchange abnormalities and worse outcomes [70]. Although not a distinct syndrome, COVID‑19 respiratory failure seems to follow a somewhat different pathophysiological trajectory [70, 71]. Immunologically, severe COVID‑19 and associated ARDS display a characteristic inflammatory signature [72]. In this context of intense systemic inflammation, high mortality, and limited early antiviral options, corticosteroids were evaluated in multiple RCTs.

Table 2 summarizes principal RCTs comparing corticosteroids with placebo in COVID‑19 [42–47]. Although the proportion formally meeting ARDS criteria was not reported, all participants had PaO₂/FiO₂ <200 mmHg and, given the high prevalence of bilateral infiltrates in COVID‑19 respiratory failure [73], most likely fulfilled the global ARDS definition [4]. Inclusion criteria, dosing regimens, and outcomes varied widely.

**Table 2:** Randomized controlled trials evaluating corticosteroids in COVID-19 ARDS

Study	Total Poulation (n) [I:Intervention (n), C:Control (n)]	Main inclusion criteria	PaO2/FiO2 (mmHg.) at inclusion, I:intervention(n) C: Control (n)	Steroid Type/dosage schedule	Duration of treatment (d)	Eficacy, Mortality Intervention (I), and Control (C)	Eficacy, Other outcomes, (Intervention vs control group)	Safety, Main adverse outcomes (Intervention vs control group)
Angus et al. 2020	384 (I = 137 and 146^†^ C = 101)	SARS-CoV-2 infection admitted to an ICU for respiratory or cardiovascular organ support	I = 137 - 149^†^ C = 138	Hydrocortisone 50 mg Q6h	7*	I = 30 and 26%^†^ C = 33% 54% OR = 1.03 and 1.10 (Bayesian analysis)	Improvement in organ support–free days within 21 days.	No differences
Dequin et al. 2020 10.1001/jama.2020.16761	149 (I = 76 C = 73)	SARS-CoV-2 infection admitted to an ICU for respiratory failure	I = 130 C = 133	Hydrocortisone 200 mg/day, continuous infusion	7	I = 15% C = 27% (*p* = 0.057) (day 21)	Not differences in treatment failure (death or requirement of respiratory support at day 21)	No differences
RECOVERY Collaborative Group. 2020	6,335 (I = 2,104 C = 4,321)	SARS-CoV-2 infection and hospitalization	NA	Dexamethasone 6 mg/day for up to 10 days	10	I = 23% C = 26% (*p* < 0.001) (day 28)^#^	↓Hospital LOS IMV requirement: reduction Renal replacement requirement: reduction	No differences
Tomazini, et al. 2020 10.1001/jama.2020.17021	299 (I = 151 C = 148)	SARS-CoV-2 infection and moderate to severe ARDS (Berlin definition)	I = 131 C = 132	Dexamethasone 20 mg/day × 5d, then 10 mg/d for up to 5 days	10	I = 56% C = 61% (*p* = 0.85) (day 28)	↑VFDs	No differences
Jamaati, et al. 2021 10.1016/j.ejphar.2021.173947		SARS-CoV-2 infection, Pao2/Fio2 100–300, bilateral infiltrates (NOT IMV)	Not rereported	Dexamethasone 20 mg/day × 5d, then 10 mg/d for up to 5 days	10	I = 64% C = 60% (*p* = 0.5) (day 28)	NO differences in: IMV requirement, improve oxygenation and SOFA score	Not reported
Prado Jeronimo, et al. 2021 10.1093/cid/ciaa1177	397 (I = 199 C = 194)	SARS-CoV-2 infection, AND supplementary oxygen requirement	I = 158 C = 156	Methylprednisolone 0.5 mg/kg twice daily for 5 days	5	I = 37% C = 38% (*p* = 0.63) (day 28)	NO differences in: IMV requirement, improve oxygenation, and hosp. LOS	No differences in nosocomial infections and insulin requirements

*Treatment could be extended up to day 28 in cases of shock and vasopressor dependence

†Two intervention groups: fixed doses up to day 7 and variable doses up to day 28 in cases of shock and vasopressor dependence

^#^Reduction in mortality among patients requiring IMV (29% vs 41%) and oxygen therapy (23% vs 26%), but NOT in those without oxygen requirements

Abbreviations. ARDS = acute respiratory distress syndrome; FiO₂ = fraction of inspired oxygen; IMV = invasive mechanical ventilation; LOS = length of stay; PaO₂ = arterial oxygen pressure; VFDs = ventilator-free days

The largest, RECOVERY, demonstrated a significant mortality reduction with dexamethasone 6 mg/day for ten days versus usual care (23% vs 26%; rate ratio 0.83; 95% CI 0.75–0.93). The effect was driven mainly by patients receiving invasive ventilation (29.3% vs 41.4%; rate ratio 0.64; 95% CI 0.51–0.81) and those requiring supplemental oxygen (23.3% vs 26.2%; rate ratio 0.82; 95% CI 0.72–0.94 [44]. Patients not requiring oxygen showed no mortality benefit and possible harm, underscoring that corticosteroids should be reserved for hypoxemic or ventilated cases.

In a comprehensive pairwise meta-analysis including both COVID-19 and non-COVID-19 ARDS, systemic corticosteroids reduced 28-day mortality overall (RR 0.82; 95% CI 0.72–0.95), with no credible evidence of effect modification according to COVID status [48]. Smaller RCTs using conventional doses (hydrocortisone 200 mg/day or dexamethasone 6–8 mg/day) [42–44, 47] or higher doses (dexamethasone 20 mg/day) [45, 46] did not individually show significant mortality differences. Pooled analyses of severe‑to‑critical COVID‑19 (including patients with ARDS) suggested a modest mortality advantage with higher doses and a reduction in the need for mechanical ventilation [74–76]. Conversely, among patients without ARDS or requiring minimal oxygen, higher‑dose corticosteroids were associated with increased mortality [74].

Based on current evidence, corticosteroids are recommended in COVID-19 patients requiring oxygen or ventilatory support, consistent with ARDS definitions. While dexamethasone 6 mg/day for ten days remains the most robustly supported regimen, higher doses (10–20 mg/day) have been explored in severe-to-critical cases and may be reasonable in selected clinical contexts.In COVID-19, systemic corticosteroids became standard anti-inflammatory therapy in hypoxemic and critically ill patients, and subsequent randomized trials evaluated additional immunomodulatory agents in this setting. Prospective meta-analyses indicate that IL-6 receptor antagonists are associated with reduced mortality, particularly when administered alongside corticosteroids [77], and network meta-analyses suggest that tocilizumab (and possibly sarilumab) in combination with corticosteroids improves survival compared with corticosteroids alone [78]. Similarly, an individual participant data meta-analysis reported that JAK inhibitors reduce 28-day mortality in hospitalized COVID-19 patients, without credible effect modification by concomitant corticosteroid use [79]. By contrast, comparable randomized evidence supporting biologic immunomodulators as adjunctive therapy in non-COVID-19 ARDS is lacking.

### Corticosteroid dosing, duration, and timing

Both biological plausibility and clinical evidence suggest that early initiation of corticosteroids may be beneficial in ARDS. Mechanistically, steroids can modulate the dysregulated inflammatory response that dominates the initial exudative phase, limiting lung injury. In trials demonstrating benefit, treatment was most commonly initiated within the first 24–48 hours after ARDS onset [17, 26, 31, 51], whereas delayed administration—particularly beyond day 14—appears not only to lack benefit but may cause harm [41].

Regarding the agent, pharmacologic considerations favor methylprednisolone due to higher pulmonary penetration and a favorable pharmacokinetic profile [12]. Some authors propose an initial loading dose followed by continuous infusion to achieve GRα saturation and sustain therapeutic levels [21]. Nevertheless, the clinical evidence shows marked heterogeneity across studies regarding corticosteroid type, dosing, and duration (Tables 1 and 2), and overlap with pneumonia and sepsis complicates interpretation. Meta‑analyses do not show significant effect modification by corticosteroid type [26, 31] Table 3.

**Table 3:** Adverse effects related to corticosteroid use in, acute respiratory distress syndrome, sepsis, and community-acquired Pneumonia*

Adverse Effects	ARDS	CAP	Sepsis	Overall
Gastrointestinal bleeding	0.75 (0.22–2.56)	1.08 (0.64–1.82)	1.10 (0.86–1.39)	1.08 (0.87–1.34)
Superinfections	0.78 (0.66–0.93)	0.96 (0.79–1.16)	1.05 (0.96–1.16)	0.97 (0.89–1.05)
Hyperglycemia	1.11 (1.01–1.23)	1.68 (1.32–2.12)	1.10 (1.06–1.14)	1.21 (1.11–1.32)
Neuromuscular weakness	1.29 (0.79–2.09)	0.14 (0.01–2.62)	1.22 (1.02–1.46)	1.22 (1.03–1.45)

Abbreviations. ARDS: Acute Respiratory Distress Syndrome. CI: confidence interval *Values expressed as relative risk (95% CI) in exposed versus not exposed to corticosteroids

Trials reporting mortality benefit most commonly employed regimens equivalent to ≥80 mg/day methylprednisolone or ≥400 mg/day hydrocortisone, administered for at least seven days [48]. Such regimens were generally associated with an acceptable safety profile [49].

A recent Bayesian dose–response network meta-analysis suggested that cumulative methylprednisolone-equivalent doses of approximately 840 mg for dexamethasone and 1,400 mg for methylprednisolone may be associated with near-maximal mortality benefit in ARDS [80]. However, these estimates derive predominantly from COVID-19 trials and model-based assumptions, and differ from prior pairwise meta-analyses that did not demonstrate a clear dose-response relationship [48]. Accordingly, the optimal cumulative dose remains uncertain.

The new ARDS definitions introduce a challenge: virtually all existing evidence—both in ARDS and in septic shock with concurrent ARDS—derives from invasively ventilated patients. Regarding non-ventilated patients who nonetheless meet ARDS criteria, the only groups for whom evidence supports a potential benefit from corticosteroids are those with severe community-acquired pneumonia treated with high-flow nasal oxygen (CAPE COD) and those with COVID-19.

**Fig. 1:**
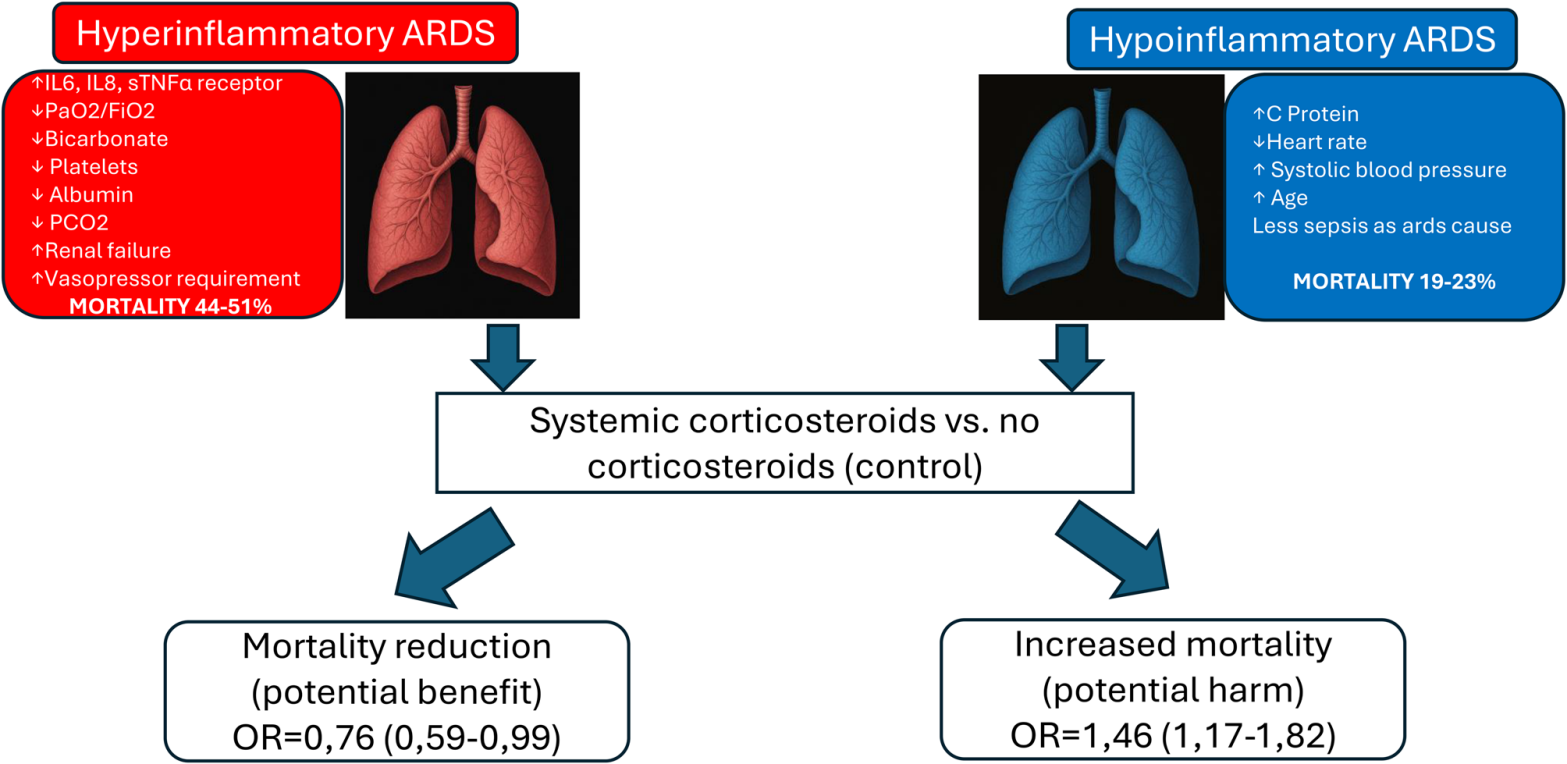
Effect of corticosteroid treatment for ARDS according to hyper- and hypo-inflammatory phenotypes

Considering these factors, the decision to initiate corticosteroids in ARDS should be grounded in clinical context and underlying etiology, guided by the body of evidence most relevant to each scenario. Rather than adopting a uniform approach, clinicians should tailor corticosteroid choice, dose, and duration to each patient’s pathophysiologic and clinical features. Our proposed algorithm (Fig. 1) translates this evidence into a pragmatic, context‑based approach emphasizing early initiation and a minimum seven‑day course with regimens equivalent to ≥80 mg/day methylprednisolone or ≥400 mg/day hydrocortisone. It is intended as a pragmatic synthesis of available evidence and should not be interpreted as a validated or society-endorsed recommendation.

### Safety of corticosteroids in the context of ARDS

Safety concerns have historically limited corticosteroid use in ARDS, particularly regarding secondary infections, gastrointestinal bleeding, neuromuscular weakness, and metabolic complications. Contemporary randomized trials and meta‑analyses have clarified the safety profile when steroids are administered at physiological or moderately supra‑physiological doses for limited durations (Table 3). Across ARDS, sepsis, and CAP, corticosteroids do not appear to increase gastrointestinal bleeding or secondary infection risk [31, 49]. In ARDS populations, steroids might even reduce superinfections—particularly ventilator‑associated or hospital‑acquired pneumonia—possibly by shortening ventilation and restoring immune balance. The most consistent adverse effects include hyperglycemia and, less frequently, hypernatremia (RR 1.59; 95% CI 1.29–1.96), both dose‑dependent and reversible. Neuromuscular weakness has historically been a major safety concern in critically ill patients receiving corticosteroids. While some contemporary pooled analyses suggest a possible increase in risk (RR 1.22; 95% CI 1.03–1.45), other evidence syntheses and guideline assessments have yielded imprecise and non-significant estimates and rate the certainty of evidence as low to very low [26, 31, 48]. Substantial heterogeneity in outcome definitions, limited systematic surveillance, and scarce long-term follow-up further limit interpretability. Accordingly, the net effect of corticosteroids on neuromuscular weakness in ARDS remains uncertain.Influenza‑associated ARDS deserves separate consideration. No RCTs have rigorously evaluated corticosteroids in this subgroup. Pooled observational analyses suggest higher mortality with corticosteroid use in influenza‑related pneumonia or ARDS, alongside increased nosocomial infection [81]. Until robust evidence becomes available, routine corticosteroid use should be avoided in influenza‑associated ARDS.

### Inflammatory subphenotypes and corticosteroid responsiveness

ARDS is increasingly recognized not as a single disease, but as a syndrome encompassing at least two reproducible clinico-biological subphenotypes—hyperinflammatory and hypoinflammatory. These phenotypes reflect distinct immune and physiological response patterns to lung injury [82–86].

The hyperinflammatory subphenotype is marked by elevated systemic inflammation, vasopressor dependence, and multi-organ dysfunction, and carries a substantially higher risk of death. In contrast, the hypoinflammatory subphenotype exhibits a more regulated host response, milder systemic involvement, and comparatively better outcomes [83].

This biological stratification helps explain why certain interventions once deemed ineffective or even harmful in unselected ARDS populations show opposing effects when viewed through a phenotypic lens. Ventilatory and fluid management strategies such as higher versus lower PEEP or restrictive versus liberal fluid balance appear to influence outcomes differently across subphenotypes, underscoring that treatment effects are not uniform but context-dependent [84, 85].

Corticosteroid therapy follows the same principle. Analyses emulating randomized trials suggest that early corticosteroid administration may be beneficial in hyperinflammatory patients—where excessive inflammation predominates—yet secondary analyses suggest a potential signal of harm in hypoinflammatory patients (OR 1.46, 95% CI 1.17–1.82), possibly reflecting maladaptive immunosuppression [82]. Recognizing these patterns highlights the need for biologically informed treatment decisions rather than uniform therapeutic application across the ARDS spectrum.

At the bedside, however, identifying subphenotypes remains challenging. Biomarker-based models combining interleukin-6, soluble TNF receptor-1, and vasopressor use achieve excellent discrimination but are not routinely feasible [84]. More accessible models integrating standard clinical and laboratory variables have been developed and externally validated in retrospective cohorts. However, no prospective randomized clinical trial has yet stratified corticosteroid therapy according to inflammatory phenotype. Current evidence suggesting differential treatment response derives from secondary analyses and trial emulations rather than prospectively phenotype-guided interventions [82].

**Fig. 2:**
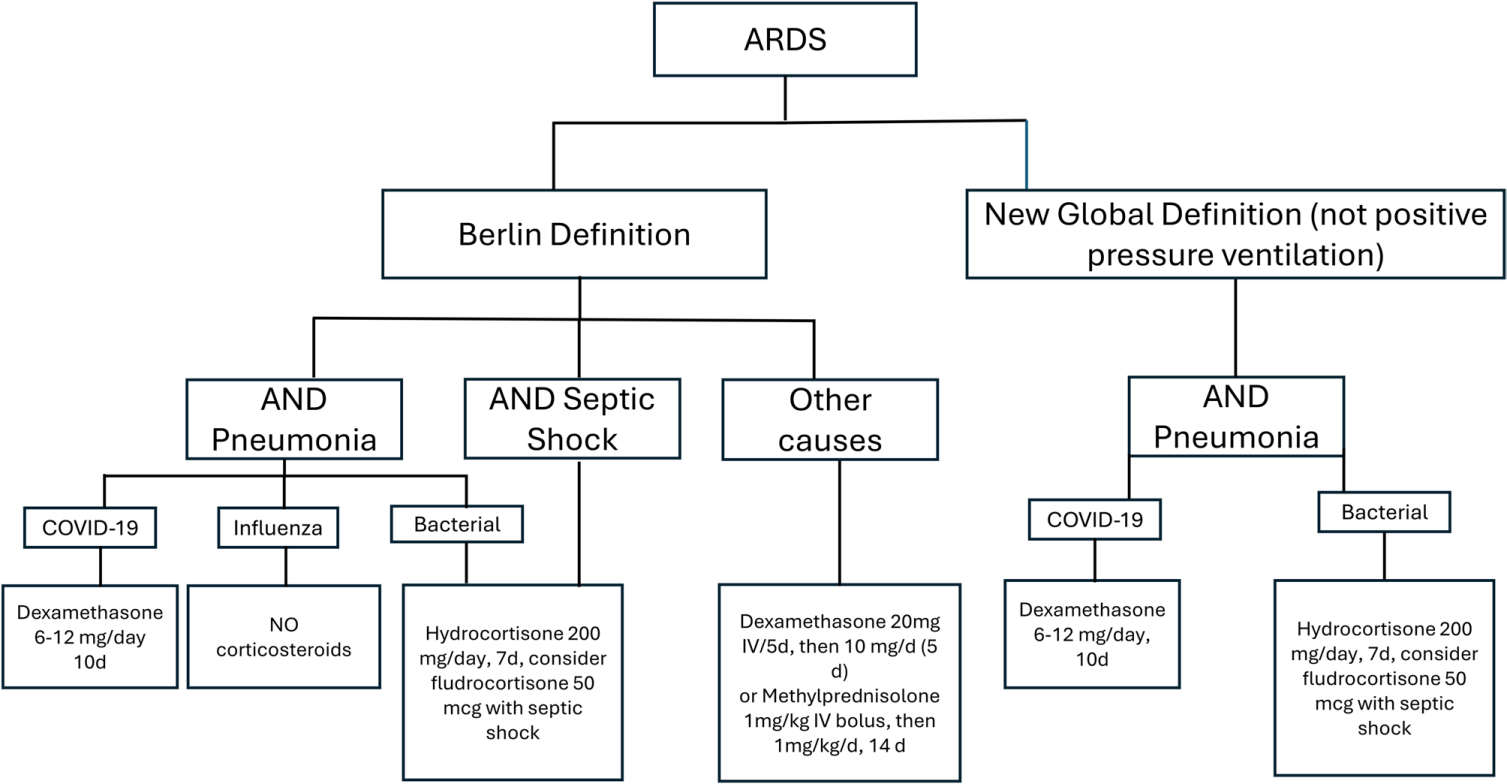
Suggested corticosteroid treatment algorithm for ARDS according to etiology and clinical context

Figure 2 summarizes this framework, depicting how inflammatory subphenotypes influence disease trajectory, response to corticosteroids, and the broader interpretation of evidence across ARDS populations. However, phenotype-based corticosteroid use should be regarded as hypothesis-generating rather than ready for routine bedside implementation.

### Guideline recommendations on corticosteroid use in ARDS

Evidence from randomized trials and meta‑analyses has been incorporated into recent guidelines. The American Thoracic Society and the Society of Critical Care Medicine, using GRADE, concluded that corticosteroids probably reduce mortality (moderate certainty) and shorten mechanical ventilation and hospital stay, while probably increasing the risk of hyperglycemia [26, 26, 30, 31]. Despite these benefits, both issued conditional recommendations, reflecting concerns about heterogeneity across trials, variability in drug type and dosing, and limited long‑term safety data. Both panels emphasized considering overlapping conditions—such as sepsis and pneumonia—where corticosteroid efficacy and safety are well established and noted that most new evidence since 2017 derives from COVID‑19 trials. The latest ESICM guidelines do not yet issue specific recommendations on corticosteroids for ARDS due to insufficient consensus [87].

Together, these guidelines converge on a key principle: corticosteroids are a reasonable option in ARDS when started early, maintained for at least seven days, and used within evidence‑based dosing ranges. Their use should be individualized, context‑specific, and aligned with the patient’s etiology, disease stage, and inflammatory profile.

### Future directions and knowledge gaps

Despite growing evidence, several gaps persist. Future studies should refine precision‑medicine approaches through enrichment strategies that identify phenotypes most likely to benefit from corticosteroids and clarify mechanisms underpinning differential responses. Underrepresented subgroups warrant targeted research, including pancreatitis‑associated ARDS, trauma, and patients who meet the global ARDS definition without positive‑pressure ventilation. Optimal dosing, timing, and tapering strategies remain uncertain and may differ across subpopulations. Prospective evaluation of long‑term outcomes, -including neuromuscular, neuropsychiatric, and infectious complications-, remains a priority.

As new immunomodulatory agents emerge, corticosteroids may serve as a foundation for combination or stepwise strategies aimed at restoring homeostasis rather than merely suppressing inflammation. Future trials should couple rigorous clinical endpoints with mechanistic investigations to determine not only whether corticosteroids work, but also in whom and how they exert benefits—bridging pathobiology with practice to inform individualized, evidence‑based care.

## Data Availability

No datasets were generated or analysed during the current study.

## Abbreviations

ARDS: Acute Respiratory Distress Syndrome
ATS: American Thoracic Society
CAP: Community-Acquired Pneumonia
CAPA: COVID-19 Associated Pulmonary Aspergillosis
CI: Confidence Interval
CIRCI: Critical Illness–Related Corticosteroid Insufficiency
COVID-19: Coronavirus Disease 2019
CT: Computed Tomography
DAD: Diffuse Alveolar Damage
ESICM: European Society of Intensive Care Medicine
FiO₂: Fraction of Inspired Oxygen
GRα: Glucocorticoid Receptor alpha
HAP: Hospital-Acquired Pneumonia
HR: Hazard Ratio
ICU: Intensive Care Unit
IL-1β: Interleukin-1 beta
IL-6: Interleukin-6
IMV: Invasive Mechanical Ventilation
LOS: Length of Stay
NF-κB: Nuclear Factor kappa B
OR: Odds Ratio
PaO₂: Arterial Oxygen Pressure
PEEP: Positive End-Expiratory Pressure
RCT: Randomized Controlled Trial
RR: Relative Risk
SARS-CoV-2: Severe Acute Respiratory Syndrome Coronavirus 2
SCCM: Society of Critical Care Medicine
TNF-α: Tumor Necrosis Factor alpha
VAP: Ventilator-Associated Pneumonia
VFDs: Ventilator-Free Days
VILI: Ventilator-Induced Lung Injury
